# Transcriptomic Profile of the Mouse Postnatal Liver Development by Single-Nucleus RNA Sequencing

**DOI:** 10.3389/fcell.2022.833392

**Published:** 2022-04-06

**Authors:** Jiangshan Xu, Shijie Hao, Quan Shi, Qiuting Deng, Yujia Jiang, Pengcheng Guo, Yue Yuan, Xuyang Shi, Shuncheng Shangguan, Huiwen Zheng, Guangyao Lai, Yaling Huang, Yang Wang, Yumo Song, Yang Liu, Liang Wu, Zhifeng Wang, Jiehui Cheng, Xiaoyu Wei, Mengnan Cheng, Yiwei Lai, Giacomo Volpe, Miguel A. Esteban, Yong Hou, Chuanyu Liu, Longqi Liu

**Affiliations:** ^1^ College of Life Sciences, University of Chinese Academy of Sciences, Beijing, China; ^2^ BGI-Shenzhen, Shenzhen, China; ^3^ Department of Biology, University of Copenhagen, Copenhagen, Denmark; ^4^ BGI College and Henan Institute of Medical and Pharmaceutical Sciences, Zhengzhou University, Zhengzhou, China; ^5^ State Key Laboratory for Zoonotic Diseases, Key Laboratory for Zoonosis Research of Ministry of Education, Institute of Zoonosis, College of Veterinary Medicine, Jilin University, Changchun, China; ^6^ Laboratory of Integrative Biology, Guangzhou Institutes of Biomedicine and Health, Chinese Academy of Sciences, Guangzhou, China; ^7^ Joint School of Life Sciences, Guangzhou Medical University and Guangzhou Institutes of Biomedicine and Health, Chinese Academy of Sciences, Guangzhou, China; ^8^ Guangdong Hospital of Traditional Chinese Medicine, Zhuhai, China; ^9^ Hematology and Cell Therapy Unit, IRCCS-Istituto Tumori‘Giovanni Paolo II’, Bari, Italy; ^10^ Bioland Laboratory (Guangzhou Regenerative Medicine and Health Guangdong Laboratory), Guangzhou, China

**Keywords:** postnatal liver development, single-nucleus transcriptomics, hepatocyte maturation, liver zonation, bile acid synthesis

## Introduction

The liver plays a vital role in maintaining the physiological homeostasis of mammals and is responsible for many biological processes, including detoxification, bile acid synthesis, glycolysis, and lipid metabolism (Ben-Moshe and Itzkovitz 2019). The liver consists of repeating anatomical units termed liver lobules, including parenchymal and non-parenchymal cells. Hepatocyte, the liver parenchymal cells, account for 60% of hepatic cells composition and 80% of liver mass (Godoy et al., 2013). While the liver non-parenchymal cells (NPCs), including bile duct cell (cholangiocyte), liver endothelial cell (LEC), hepatic stellate cells (HSC), Kupffer cell, and other immune cell populations, account for the remaining 20% of liver mass (MacParland et al., 2018; Aizarani et al., 2019). When the cell function is compromised or its composition becomes abnormal, it can cause many diseases, such as fatty liver disease, cirrhosis, and hepatocellular carcinoma (Saviano et al., 2020).

During embryonic development, a few cells from the endoderm begin to specialize into hepatoblasts around embryonic day (E) 8.5 - E9.0 in mouse embryos (Tremblay and Zaret 2005). By E10.5, hepatoblast, LEC, and HSC start to organize into primitive sinusoidal capillaries (Gordillo et al., 2015). Finally, hepatoblast begin to differentiate into hepatocyte and cholangiocyte approximately at E13.5 (Roskams and Desmet 2008; Yang et al., 2017). In those developmental processes, several transcription factors and signaling pathways, including *Hhex*, hepatocyte nuclear factor 4α (Hnf4α), fibroblast growth factors (FGFs), bone morphogenetic proteins (BMPs), Wnt/β-catenin, and Hippo pathway play essential roles (Bort et al., 2006; McLin et al., 2007; Negishi et al., 2010; Alder et al., 2014; Wang et al., 2015; Ober and Lemaigre 2018). Until E16.5, the liver is the main hematopoietic organ in the body, then gradually becomes a metabolic organ (Zaret 2002). After birth, both the nutrient metabolism and the immune system in the liver will undergo significant transformations in response to drastic changes, such as the primary source of energy switching from glucose in the cord blood to lipids in breast milk (Ehara et al., 2015). As a result, metabolic pathways, including fatty acid β-oxidation, gluconeogenesis, and *de novo* lipogenesis, are upregulated in the neonatal liver (Perichon and Bourre 1995; Sekine et al., 2007). After a short period of adaptation, hepatocytes begin to proliferate and differentiate rapidly around postnatal day 3 (P3), as a result of increased β-catenin signaling (Apte et al., 2007). By P7, the liver lobule structure has been formed (Ober and Lemaigre 2018), becomes more intact, and performs several functions, including xenobiotic metabolism, steroid metabolism, and bile acids biosynthetic (Li et al., 2009; Cui et al., 2012).

In recent years, single-cell transcriptome sequencing has been developing rapidly and has been applied in many research fields, such as cell atlas construction, as well as studying embryo development and disease pathogenesis (Granja et al., 2019; Tang et al., 2019; Cao et al., 2020; Trevino et al., 2021). In terms of liver development, single-cell transcriptome sequencing has been used to elucidate the origin of hepatoblasts, the differentiation trajectory of hepatoblasts and NPCs, and the interaction among different cell types (Popescu et al., 2019; Lotto et al., 2020; Mu et al., 2020; Wang et al., 2020). However, few single-cell transcriptomic studies on liver development in mice after birth. A robust and comprehensive bulk RNA sequencing study was performed to characterize the liver development spanning E12.5 to postnatal week 8, which found that many important liver metabolic functions are acquired after birth (Gong et al., 2020). However, bulk RNA sequencing cannot distinguish the gene expression of different cell types and does not provide information about cell-cell interaction and microenvironment composition, thus calling for a more systematic single-cell transcriptome study of postnatal liver development. Moreover, single-nucleus RNA sequencing (snRNA-seq) has an obvious advantage over single-cell RNA sequencing (scRNA-seq) in detecting multiple cell types without any bias (Zeng et al., 2016; Lake et al., 2017; Bakken et al., 2018; Ding et al., 2020). This is crucial for liver tissue because hepatocytes are prone to cell death during liver single-cell isolation, and obtaining HSCs and cholangiocytes remains quite challenging. In addition, it could lead to skewed cell types ratio in scRNA-seq datasets (Donne et al., 2020; Brazovskaja et al., 2021; Guilliams et al., 2022).

For this study, we performed snRNA-seq to profile 82,967 nuclei from four key time points of postnatal murine liver development (P0, P3, P7, P14). We identified 28 clusters of hepatic cell types and analyzed the dynamic changes in cells composition and functions and the hepatocyte differentiation trajectories during this process. Interestingly, we found two HSC subtypes specifically expressing some markers of LECs or Kupffer cells. In addition, the ligand-receptor interaction and transcription factor regulative activity analysis significantly increased the reliability of the new cell types.

## Materials and Methods

### Sample Collection

All mice used in this study were in C57BL/6 background. The Institutional Review Board approved the use of mice in relevant experimental studies on the Ethics Committee of BGI (Permit No. BGI-IR20210903001). Four neonatal mice from different time points (P0, P3, P7, P14) after birth were purchased from Jiangsu Ailingfei Biotechnology Co. LTD. and used in this study. Mice were transported to the Guangzhou Institute of Biomedicine and Health (GIBH) of the Chinese Academy of Sciences, where GIBH colleagues helped with tissue dissection. Liver tissues were harvested, resected, and snap-frozen in liquid nitrogen. The dissected mouse liver tissues were transported on dry ice to BGI-Shenzhen and were immediately stored in a liquid nitrogen tank.

### Nuclei Isolation From Frozen Tissues

Nuclei were isolated from frozen mouse liver tissue according to a published nucleus extraction method (Corces et al., 2017). All the subsequent procedures were performed on ice. Briefly, each frozen liver tissue was cut into pieces and transferred to a prechilled 2 ml tissue Dounce homogenizer (Sigma, #D8938-1SET) with 2 ml of ice-cold homogenization buffer [500 mM sucrose (Sigma, #69293), 1% BSA (Sigma, #V900933-100G) in nuclease-free water, 20 mM Tris pH 8.0 (Sigma, #T2694-1L) 50 mM KCl (Sigma, #P5405), 10 mM MgCl_2_ (Sigma, #2670-100g), 0.1% NP-40 (Invitrogen, #FNN0021), 1 × protease inhibitor cocktail (Thermo Scientific, #87786), 0.1 mM DTT (Sigma, #646563), and 0.12 U/µl RNasin Plus (Promega, #N2115)]. The tissue was incubated on ice for 5 min and homogenized by 25 strokes of the loose Dounce pestle, after which the homogenate was filtered through a 70 μm cell strainer (Sigma, #CLS431752-50EA). Next, the filtered homogenate was further homogenized by 25 strokes of the tight pestle to release nuclei, then filtered through a 40 μm cell strainer (Sigma, #CLS431750-50EA) into a 15 ml centrifuge tube and centrifuged at 500 g for 5 min. The sediment was resuspended in 1.5 ml blocking buffer containing 1 × phosphate buffer saline (PBS, Thermo Fisher Scientific, #10010049), 1% filtered sterilized BSA, and 0.2 U/ml RNasin Plus by pipetting up and down gently and centrifuged at 500 g for 5 min. The previous step was repeated once. The nuclei were resuspended in 0.04% BSA of PBS, then counted by DAPI (Beyotime, #C1006) staining and diluted to a concentration of 1,000 nuclei/μl.

### snRNA-Seq Library Preparation and Sequencing

The single-nucleus RNA-seq libraries were prepared as previously described (Liu et al., 2019) with DNBelab C Series High-throughput Single-Cell RNA Library Preparation Kit (MGI, 940-000047-00). Briefly, the single cell suspension, barcoded mRNA capture beads, and droplet generation oil were loaded into the corresponding reservoirs on the chip for droplet generation. The droplets were placed at room temperature for 20 min and then broken and collected by the bead filter. The beads pellet was resuspended with 100 μl RT mix. The mixture was then thermal cycled as follows: 42°C for 90 min, 10 cycles of 50°C for 2 min, 42°C for 2 min. The PCR master mix was added to the beads pellet, and thermal cycled as follows: 95°C for 3 min, 15 cycles of 98°C for 20 s, 58°C for 20 s, 72°C for 3 min, and finally 72°C for 5 min. Amplified cDNA was purified using 60 μl of DNA clean beads (VAZYME, #N411-03). According to the manufacturer protocol, the cDNA was subsequently fragmented by NEBNext dsDNA Fragmentase (New England Biolabs, #M0348L). The indexed sequencing libraries were constructed and sequenced using the DIPSEQ T10 sequencer at the China National GeneBank.

### Raw Sequencing Data Processing

The read structure was paired-end with read 1 covering 30 bases in which the 1st–20th bases were cell barcodes, and the 21st–30th bases were unique molecular identifier (UMI) sequences. The read 2 contains 100 bp of transcript sequences. The PISA software (https://github.com/shiquan/PISA) was used to parse raw reads into FASTQ+ format based on the library structure and correct cell barcodes with the allow list if the hamming distance is equal or lower than one. The reformed reads were aligned to reference genome GRCm38 (mm10) by using STAR (Dobin et al., 2013) software. SAM files were transformed into BAM files and annotated with a reference gene set using *PISA* software. The UMIs in reads with the same cell barcode and gene annotation containing 1 bp mismatch were corrected to the most supported one. Then, we filtered the empty droplets by using R package DropletUtils (Lun et al., 2019). A final cell-x-gene matrix was generated by PISA too. The SoupX (Young and Behjati 2020) software was harnessed to remove the influence of ambient RNA.

### Doublet Filtering, Batch Effect Eliminating, and Cell Clustering

The final cell-x-gene matrix was introduced into the Seurat (v4.0.0) package to create a Seurat object followed by normalization, scaled, and dimensionality reduce by the CreateSeuratObject, NormalizeData, FindVariableFeatures, ScaleData, and RunPCA functions in turn with default parameters (Hao et al., 2021). Then, we performed the DoubletFinder (v2.0.3) package to filter the doublet cells (McGinnis et al., 2019). The CCA algorithm implemented by the FindIntegrationAnchors and IntegrateData functions in Seurat was used to integrate all the filtered objects from each sample. Finally, taking advantage of the FindNeighbors and FindClusters functions, we divided all cells into 20 clusters covering the most common hepatic cell types.

### Hepatocyte Trajectory Inference

About 10,000 hepatocytes were extracted from the integrated Seurat object and introduced into Monocle 2 (v2.18.0) by the as.CellDataSet function (Trapnell et al., 2014). Then, we reduced the dimensions by using the reduceDimension function with the method of DDRTree. At last, we inferred the pseudo-temporal cell transition process and split cells into 13 states with the orderCells function. Finally, the genes changing along pseudotime were identified using the differentialGeneTest function with the formula “∼sm.ns (Pseudotime)”.

### Regulatory Network Inference

To investigate the possible regulatory network intra- or inter-cell, we performed transcript factor regulatory analysis and ligand-receptor interaction analysis by using the pySCENIC (v0.11.2) (Van de Sande et al., 2020) package and CellChat (v1.1.0) (Jin et al., 2021) package, following the tutorials respectively. The transcript factors regulative activity matrix, exported from the pySCENIC pipeline, was inserted to the Seurat object as a new assay, and the cell type-specific regulons were identified by using the FindAllMarkers function of Seurat and visualized by the DimPlot function of Seurat. In addition, the netVisual_circle function visualized the ligand-receptor-interaction results.

### Visualization

The marker gene dot plot and Uniform Manifold Approximation and Projection (UMAP) were visualized by the DotPlot and DimPlot functions of Seurat, respectively. The bar plot of GO term enrichment was visualized by the barplot function of the clusterProfiler package (v3.18.1) (Yu et al., 2012). The plot_cell_trajectory function of Monocle 2 visualized the trajectory embedding dot plot. Other dot plots, box plots, violin plots, bar plots, and stream plots were visualized by ggplot2 package (v3.3.3) with the color palette of ArchR package (v1.0.1) (Hadley 2016; Granja et al., 2021).

## Results

### snRNA-Seq Data Quality Control

**FIGURE 1:**
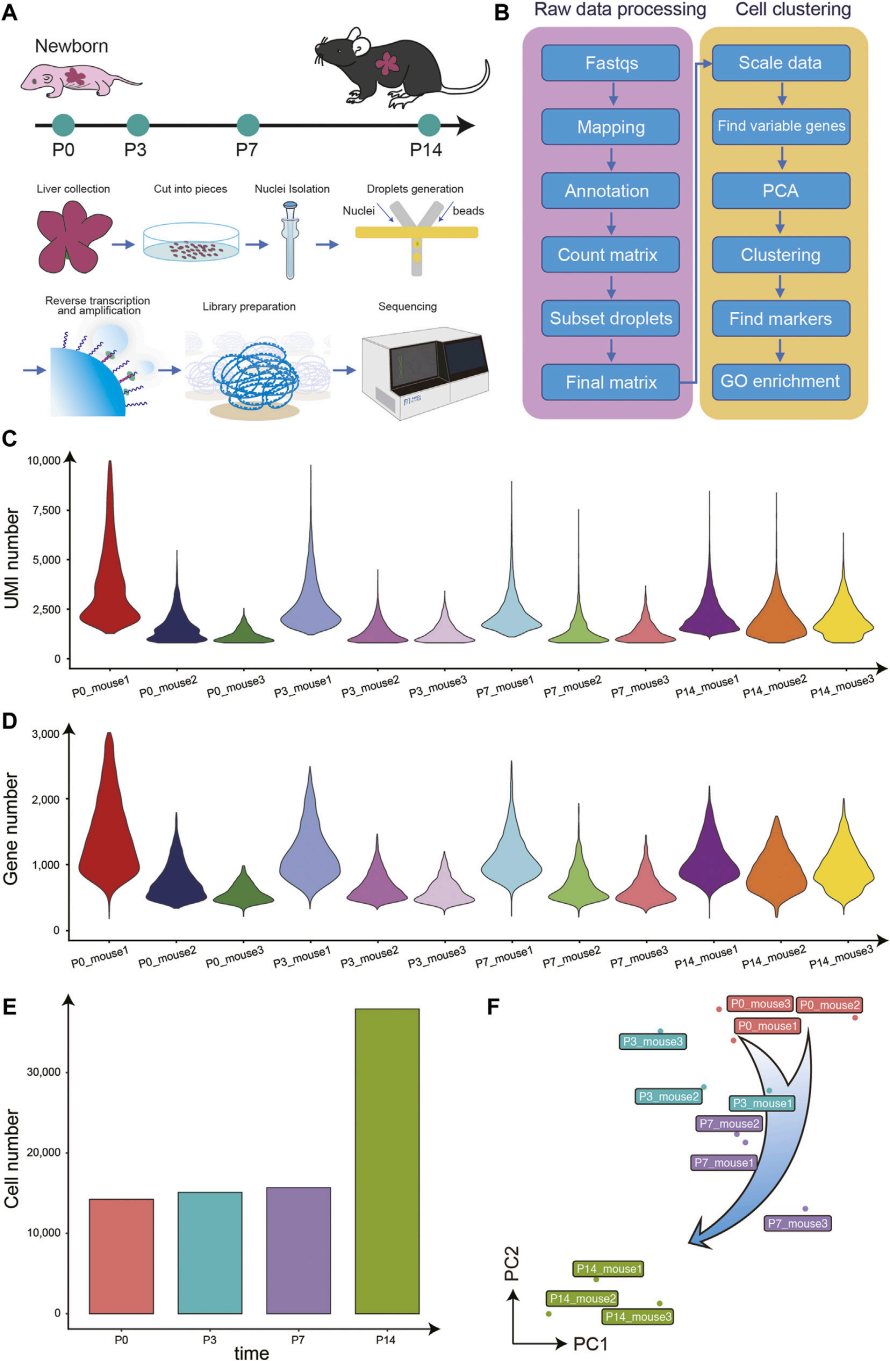
Overview of the experimental design, data analysis workflow, and snRNA-seq data quality control. **(A)** The mouse liver samples of four different time points after birth were collected for snRNA-seq profiling. **(B)** The analysis workflow for snRNA-seq profiles. **(C)** The violin plot for the UMIs number of each library. **(D)** The violin plot for the genes number of each library. **(E)** The histogram for the cell numbers each time point. **(F)** The PCA analysis for all libraries.

To generate an overview of postnatal liver development at the single-cell resolution, we performed snRNA-seq on the liver of mice at P0, P3, P7, P14, and there are three biological replicates at each time point (Figure 1A). First, we evaluated the raw sequencing data quality through several parameters, including the total reads number, the fraction of reads with a valid barcode, and Q30 of reads and barcodes. Around 36,370 million of the raw reads were filtered into a total of 26,610 million clean reads (Supplementary Table 1). The average bases Q30 in reads and barcodes were 87.6 and 92.0%, respectively. Following the raw data preprocessing, we performed the data parsing, reads mapping, alignment annotation, and matrix counting. To further eliminate the effect of doublets in snRNA-seq data, we performed one round of doublet filtering (Figure 1B).

In total, 82,967 single nucleus transcriptomes from the 39 snRNA-seq libraries passed quality control, with a median number of 2,254 UMIs and 1,005 genes per cell (Figures 1C–E). Besides, the principal component analysis (PCA) showed that samples from the same time point clustered together and the P14 libraries were far away from the libraries of the other three time points, consistent with previous reports (Gong et al., 2020; Chembazhi et al., 2021), indicating the high library quality and repeatability (Figure 1F).

### Cell Types Composition During Postnatal Liver Development

**FIGURE 2:**
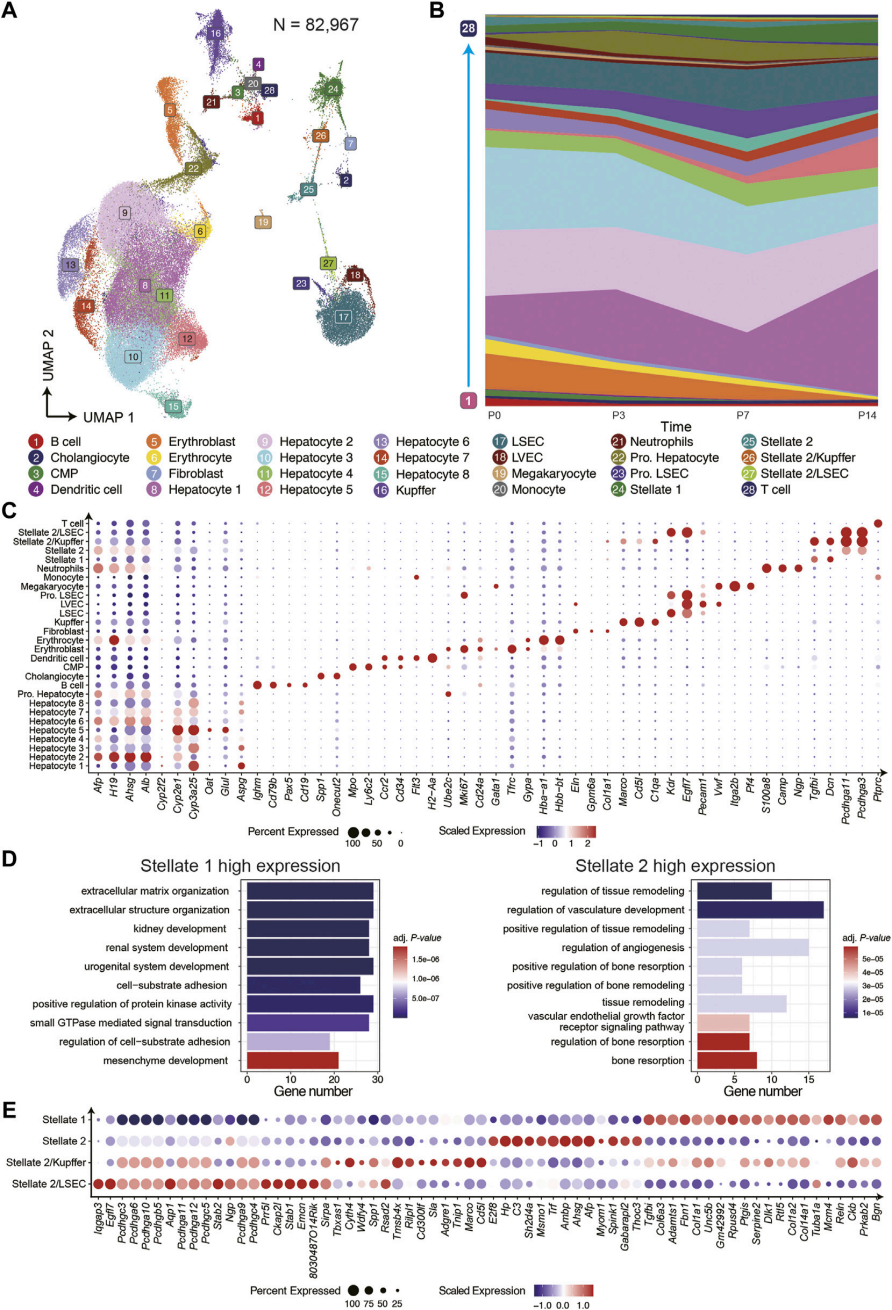
The cell type composition of postnatal liver development. **(A)** The UMAP of snRNA-seq data displayed 28 clusters in four time points. **(B)** The cell composition was dynamic at four time points. **(C)** The dot plot for cell-type-specific genes expression in 28 clusters. **(D)** The GO term enrichment analysis of differential genes of two hepatic stellate clusters. **(E)** Highly expressed genes in hepatic stellate cell subtypes

To investigate the cells composition and the functional diversity of different cell types during postnatal liver development at single-cell resolution, we integrated all snRNA-seq data from four time points and clustered them into 28 clusters with eliminated batch effect by using Seurat (Figure 2A; Supplementary Figure 1). We further annotated the 28 clusters to the known liver resident cell types based on the distinct expression of canonical marker genes and analyzed the changes of cell composition at different time points. (Figures 2B,C; Supplementary Figure 2).

For hepatocytes, we identified nine subtypes at different stages of differentiation. The hepatocytes 2, 3, and 6 were immature cells in the early stage of postnatal liver development. These cells highly expressed *Afp*, *H19*, and *Ahsg*, which were essential factors for hepatocyte differentiation and tumorigenesis in hepatocellular carcinoma (HCC) (Lazarevich 2000; Kalabay et al., 2007; Pope et al., 2017). The hepatocytes 1, 4, 5, 7, and 8 were characterized by the high expression of genes related to liver metabolic functions, and the proportion of these cells increased gradually during postnatal liver development. The genes exclusively expressed in hepatocyte 1 contained many periportal exhibited features in the adult mouse liver, such as *Apsg*, *Sds*, and *Cyp2f2* (Halpern et al., 2017; Ben-Moshe et al., 2019). On the contrary, the genes highly expressed in the hepatocyte 5 contained more pericentral features of the liver zonation, such as *Glul*, *Cyp2e1*, and *Oat*. The Pro. Hepatocyte group consisted of proliferating hepatocytes and highly expressed proliferation genes, such as *Mki67* and *Ube2c*, with those cells being more abundant in P3 and P7. The Cholangiocytes were detected at all time points, specifically expressing *Spp1* and *Onecut2*. And the latter one may be a new marker gene.

Regarding the liver resident non-parenchymal cells, including LEC, HSC, Kupffer cell, we identified many cell subtypes and compared their cell proportion at all time points. Besides, the Kupffer cells increased significantly at P3 and P7 but decreased sharply at P14, suggesting more immune challenges to respond to drastic environmental changes after birth. On the other hand, three LEC clusters, including liver sinusoidal endothelial cell (LSEC), proliferating endothelial cell, and liver vessel endothelial cell (LVEC), that specifically expressed *Vwf* were discovered*.* For mesenchymal cells, we identified two HSCs subtypes and fibroblast. The fibroblast distinctively expressed *Eln*, *Col1a1,* and *Gpm6a*. In addition, both the HSC subtypes expressed classical marker genes *Dcn*, *Reln*, and *Fgfbi*.

Interestingly, the stellate 2 exclusively expressed many genes of the protocadherin family, indicating a critical role in establishing the specific cell-cell connections (Hirayama et al., 2012). Next, we compared the differential expressed genes (DEGs) of these two groups of HSCs and performed the Gene Ontology (GO) terms enrichment analysis. We found that stellate 1 highly expressed genes related to extracellular matrix organization and cell adhesion, while the DEGs of stellate 2 were mainly enriched in signaling pathways related to angiogenesis and hypoxia response (Figure 2D). The cluster stellate 2 displayed a transitional pattern in UMAP spanning LSEC to Stellate 1, indicating its composition complexity. For a deeper understanding of stellate 2, we regrouped it into three subgroups and identified the marker genes in each population (Figure 2E). There were two new heterogeneous cell types in these three subgroups, one of which expressing *Cd5l* and *Marco* was annotated as Stellate 2/Kupffer, which was recently mentioned by a published paper (Liang et al., 2022). Another heterozygous group expressing *Kdr* and *Egfl7* was annotated as Stellate 2/LSEC. Further, we analyzed the ligand-receptor interactions between stellate and other cell types and found the multiplex functions of these two heterogeneous subgroups (Supplementary Figure 3). For example, the VEGF signaling pathway is enriched in Stellate 2/LSEC cell and LSEC; the AGT signaling pathway is more enriched in Stellate 2/Kupffer cell and Kupffer. These results further suggest that the newly discovered cell types are reliable. Moreover, we found the CDH signaling pathway enriched among hepatocytes, proliferating hepatocytes, and stellate 2, indicating the effect of stellate 2 on hepatocyte proliferation.

We also identified many NPCs cell types of hematopoietic origin that can be grouped into three main lineages: lymphoid, myeloid, and erythroid. We observed several clusters of B cells at different stages of differentiation, as characterized by the expression of genes involved in regulating B cells differentiation and maturation, such as *Pax5*, *Cd79b*, and *Cd19*. In our study, T cells were also found expressing *Ptprc* (*Cd45*). The myeloid cells identified in our datasets consisted of common myeloid progenitor (CMP), neutrophils, monocytes, and dendritic cells, expressing classic marker genes, such as *Mpo*, *S100a8*, *Ccr2*, and *H2-Aa*, respectively. The erythroid lineage contained erythroblast and immature erythrocyte, both of which highly expressed *Hba-a1* and *Hbb-bt*. The erythroblast, also highly expressed *Gypa* and *Gata1* are a critical determinant of erythrocyte differentiation (Cao et al., 2020). In addition, we observed the expression of genes associated with platelet formation, such as *Pf4* and *Plek*, in megakaryocytes (Italiano and Shivdasani 2003) (Figure 2C; Supplementary Figure 2). Almost all these developing hematopoietic cells except for T cells gradually decreased during liver development and eventually disappeared by P14, which is consistent with the conclusion of a paper published in the journal of Hepatology in 2018 (Nakagaki et al., 2018).

### Cellular Trajectory of Hepatocyte Differentiation

**FIGURE 3:**
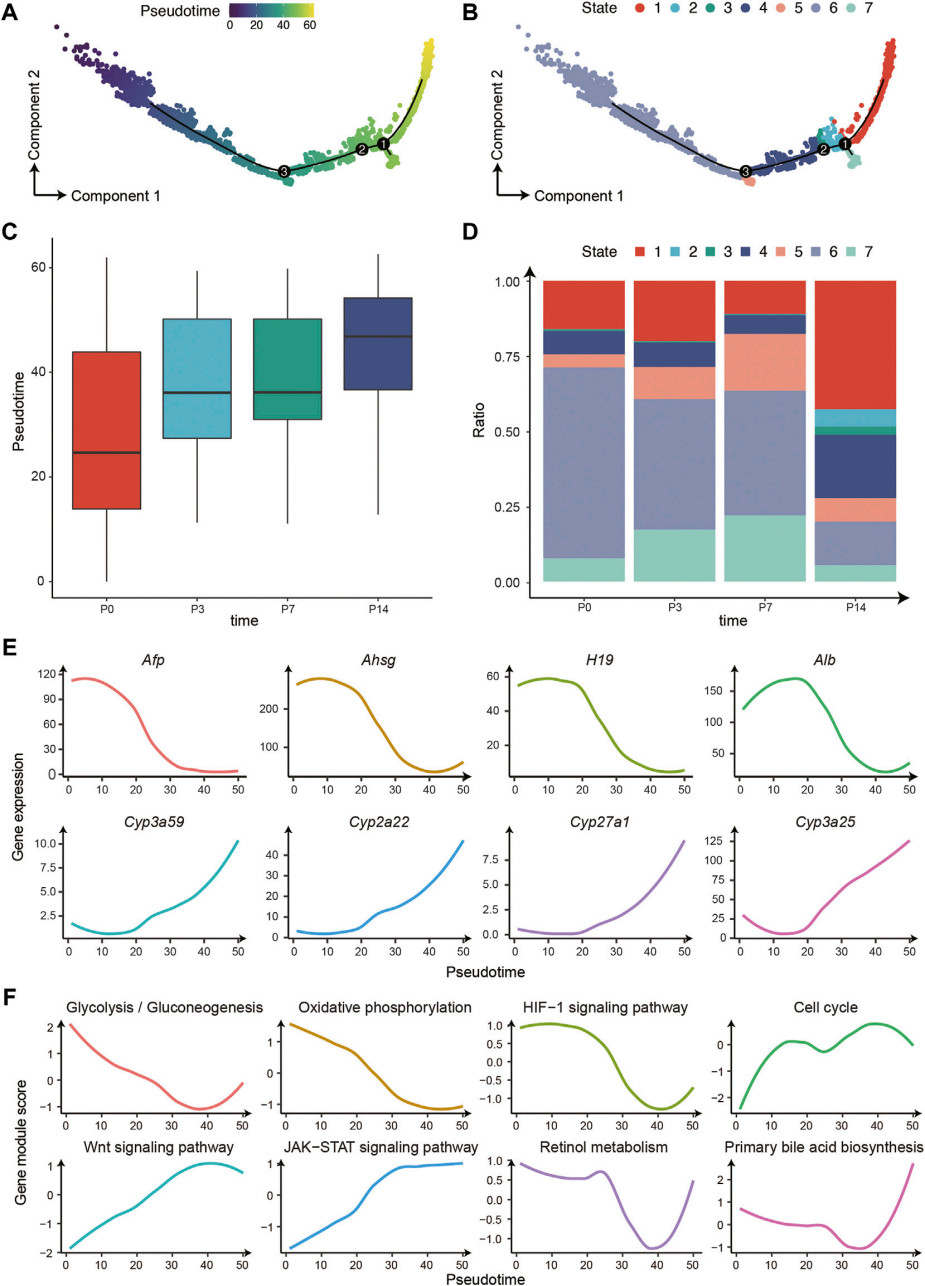
The differentiation trajectory of hepatocytes. **(A)** The trajectory of hepatocyte development from P0 to P14 with Monocle 2. The colors from blue to yellow indicate an increased pseudotime. **(B)** The trajectory is the same as **(A)**. The colors represent 7 different states. **(C)** The boxplot for the pseudotime distribution in four time points. **(D)** The histogram displayed the hepatocyte numbers of 7 states in four time points. **(E)** The gene expression changes patterns along the trajectory. **(F)** The pathway changes patterns along the trajectory.

To further characterize the hepatocyte differentiation process and the liver metabolic function dynamics, we used Monocle 2 to establish the developing trajectory and calculated the pseudotime for each nucleus (Figure 3A). There was a clear differentiation trajectory from left to right, consisting of three branching points that divide all hepatocytes into 7 states (Figure 3B). In addition, the pseudotime distribution and cell state composition across four time points displayed great coordination, further confirming the validity of our trajectory analysis (Figures 3C,D). Furthermore, we analyzed the variation of a series of essential genes related to liver development and found that the expression of marker genes of immature hepatocytes decreased gradually along the trajectory, such as *Afp*, *Ahsg*, and *H19*. In contrast, the expression of liver metabolism-related genes increased significantly, such as *Cyp3a59*,*Cyp2a22*, *Cyp27a1*, and *Cyp3a25* (Figure 3E). Therefore, the trajectory reconstructed the procession of hepatocyte maturation.

Moreover, we investigated the pathway changes along the differentiation trajectory of hepatocytes (Figure 3F). The dynamic of these pathways can be further divided into three main categories: 1) The decreased pathways including glycolysis/gluconeogenesis, oxidative phosphorylation, and *HIF-1* signaling pathway, possibly owing to the changes of oxygen and nutrients supply after birth (Bohme et al., 1983); 2) Pathways that increased in P3 and P7 and decreased in P14, including cell cycle and WNT signaling pathway, which regulates the proliferative capacity of hepatocytes; 3) The increased pathways including the JAK-STAT signaling pathway, retinol metabolism, and bile secretion, that are mainly involved in the acquisition of metabolic function of hepatocytes post birth.

### Cell Type-Specific Transcription Factor Activity Analysis

Finally, we investigate the essential regulatory genes in the mouse liver development process after birth. We performed single-cell regulatory network inference and clustering (SCENIC) analysis (Aibar et al., 2017). And we captured 12 cell type-specific transcription factors (Regulon) that may significantly affect the fate of hepatic cells and liver function in adults (Supplementary Figure 4). For instance, FOXA2 (Hepatocyte Nuclear Factor 3-Beta, HNF3B), HNF4A, and SOX9 are essential in determining the differentiation of hepatoblast (Gordillo et al., 2015), had higher regulative activity in hepatocytes or cholangiocytes. Besides, NR1H4, NR1I2, and CEBPB can affect multiple liver functions, including bile acid synthesis and transport, detoxification, and regeneration (Milnes et al., 2008; Jakobsen et al., 2013; Haeusler et al., 2016) had different regulative activity in different hepatocyte subgroups, implying their various functions in hepatocyte development. For the liver non-parenchymal cells, we found that: TCF4 is enriched explicitly in LEC; MAFB is enriched explicitly in Kupffer cell; GATA6 and FOXF1 are enriched in fibroblast and stellate, 1 respectively. Mainly TFAP4 was extremely cell type-specific in stellate 2, a gene that promotes tumorigenic capability and activates the Wnt/β-catenin pathway in hepatocellular carcinoma in previous studies (Zhao and Duncan 2005; Song et al., 2018).

## Conclusion

In summary, we profiled the single-nucleus transcriptome of mouse postnatal liver development at four time points, identified 28 different hepatic cell types, and investigated the dynamic of cells composition. Interestingly, we identified a new subtype of hepatic stellate cells that exclusively expressed many genes of the protocadherin family, such as *Pcdhg3* and *Pcdhg11*, which may be of great importance in the establishment of specific cell-cell connections. Furthermore, by regrouping and annotation, we obtained two new heterogeneous cell subtypes in stellate 2, respectively expressed markers of LEC and Kupffer cell, including *Egfl7*, *Kdr*, *Cd5l*, and *Macro*. Moreover, the CellChat analysis showed that the CDH signaling pathway only enriched among hepatocyte clusters and stellate 2, which may influence the proliferation of hepatocyte. For hepatic parenchyme cell, we identified several immature hepatocyte subtypes and cholangiocyte, respectively highly expressed *Afp*, *Ahsg*, *Spp1*, and *Onecut2*. But we do not find the liver stem cell population (hepatoblast) by checked the expression levels of classic marker genes, such as *Dlk1*, *Nope*, *Cd24a*, *Prom1* and *Epcam*, which is consistent with the known fact that hepatoblast differentiation mainly occurs from E13.5 to E18.5 during mouse embryonic development (Miyajima et al., 2014; Gordillo et al., 2015; So et al., 2020; Wang et al., 2020). Consider that two important recent studies by lineage tracing in mice have reported that there are no adult stem cells in the liver, but that there are differences in the regional hepatocyte proliferation of the liver lobule (He et al., 2021; Wei et al., 2021), so we think that there are only immature hepatic cells in the mouse liver after birth according to the data of this study, which requires further experimental verification by assays including chimera or organoid (Leeb and Wutz 2011; Leeb and Wutz 2012; Takebe et al., 2017; Li et al., 2018). Such immature hepatic cells during liver development are very important for the study of expanded culture of hepatocytes *in vitro*, organoid and disease models, because they have some similar characteristics to hepatocyte progenitor cells and strong cellular plasticity (Li et al., 2020). In addition, we found that several signaling pathways changed along the hepatocytic differentiation trajectory, such as glycolysis/gluconeogenesis, oxidative phosphorylation, bile secretion, JAK-STAT, WNT signaling pathway, among others. Finally, we identified key transcription factors activity enriched in different cell clusters, including FOXA2 (HNF3B), HNF4A, and SOX9. Both FOXA2 and HNF4A are pioneer hepatocyte transcription factors for the differentiation of embryonic stem cell from the foregut endoderm into hepatoblast during embryonic development, which activated liver-specific genes such as *Alb* and *Ttr* (Olsen and Jeffery 1997; Alder et al., 2014; Gordillo et al., 2015). SOX9 significantly affect hepatoblast differentiated into cholangiocyte and has been used as a marker gene for liver progenitor cell in certain conditions for a period of time (Antoniou et al., 2009; Tarlow et al., 2014). In a word, this dataset will be a valuable resource to understand the fundamental biological events in postnatal liver development, such as the differentiation process of hepatocytes, angiogenesis, and the postnatal acquisition of metabolic functions.

## Data Availability

The datasets presented in this study can be found in online repositories. The names of the repository/repositories and accession number(s) can be found below: BioProject: PRJNA790690 and China National GeneBank DataBase (CNGBdb) Project accession : CNP0002461.

## Supplementary Material

Supplementary Figure 1 The UMAP of snRNA-seq data shows 20 clusters in four time points.

Supplementary Figure 2 Cell-type-specific marker genes expression visualized by UMAP.

Supplementary Figure 3 HSCs related ligand-receptor interaction network.

Supplementary Figure 4 Cell-type-specific transcription factors regulation activity visualized by UMAP.

Supplementary Table 1 snRNA-seq raw data statistics.
