# Supplementary figures and images for: Transcriptomic Profile of the Mouse Postnatal Liver Development by Single-Nucleus RNA Sequencing
Source: Front Cell Dev Biol. 2022 Apr 6;10:833392. doi: 10.3389/fcell.2022.833392 (PMC9019599; doi:10.3389/fcell.2022.833392)

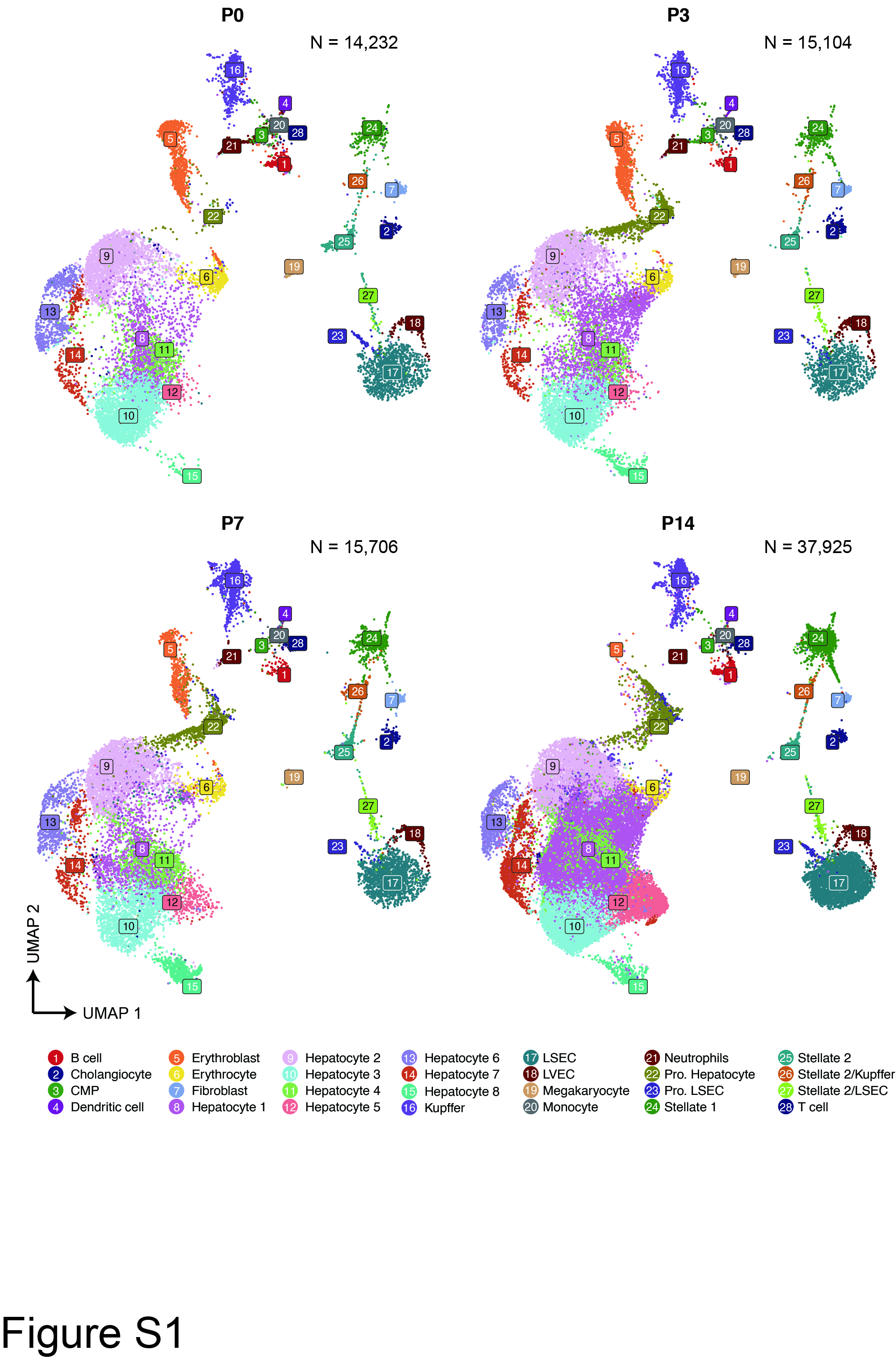

Supplement: Supplementary file 1 [file DataSheet1.ZIP › Additional files/FigureS1_noborder.tif]

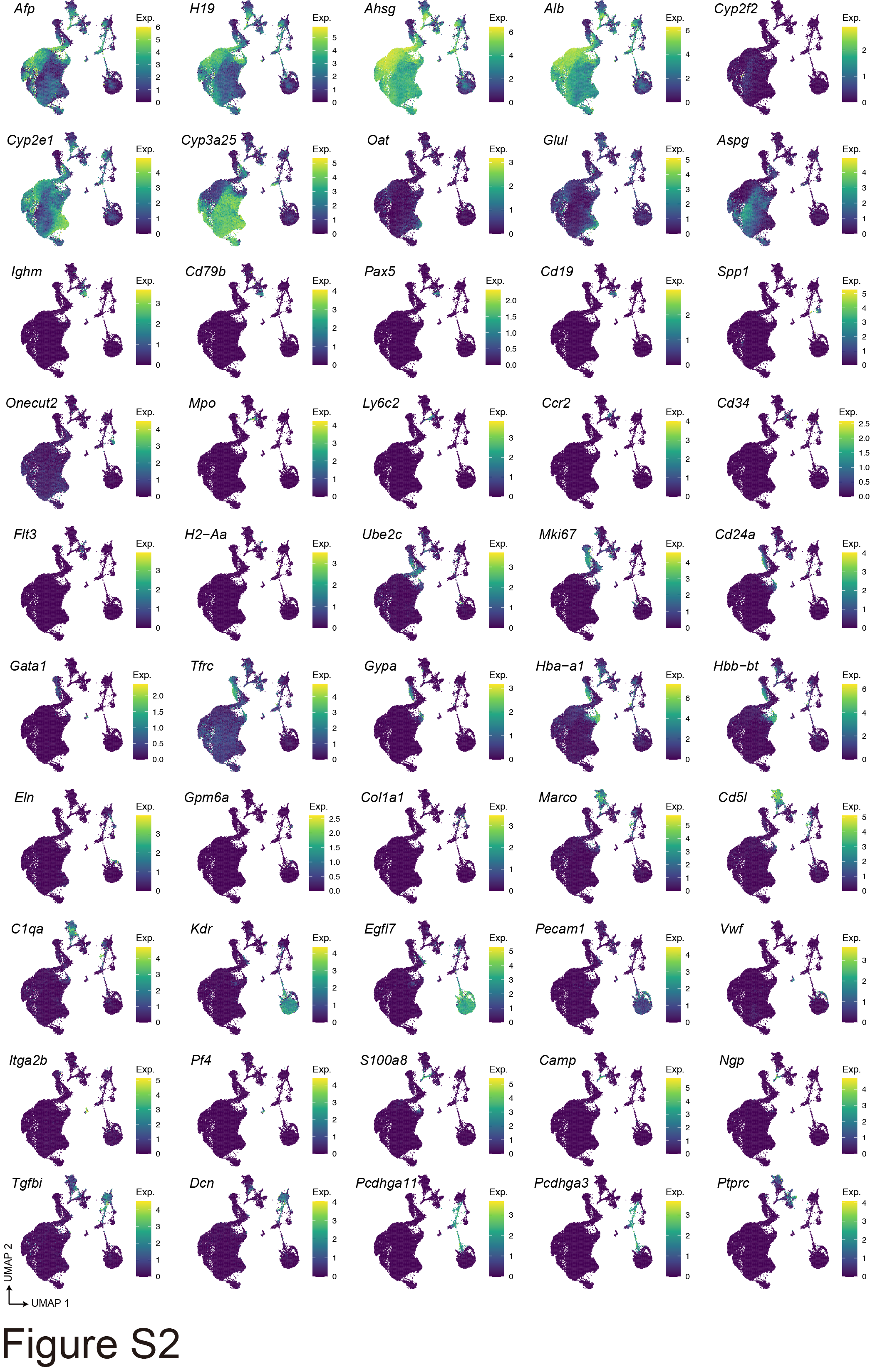

Supplement: Supplementary file 1 [file DataSheet1.ZIP › Additional files/FigureS2_noborder.tif]

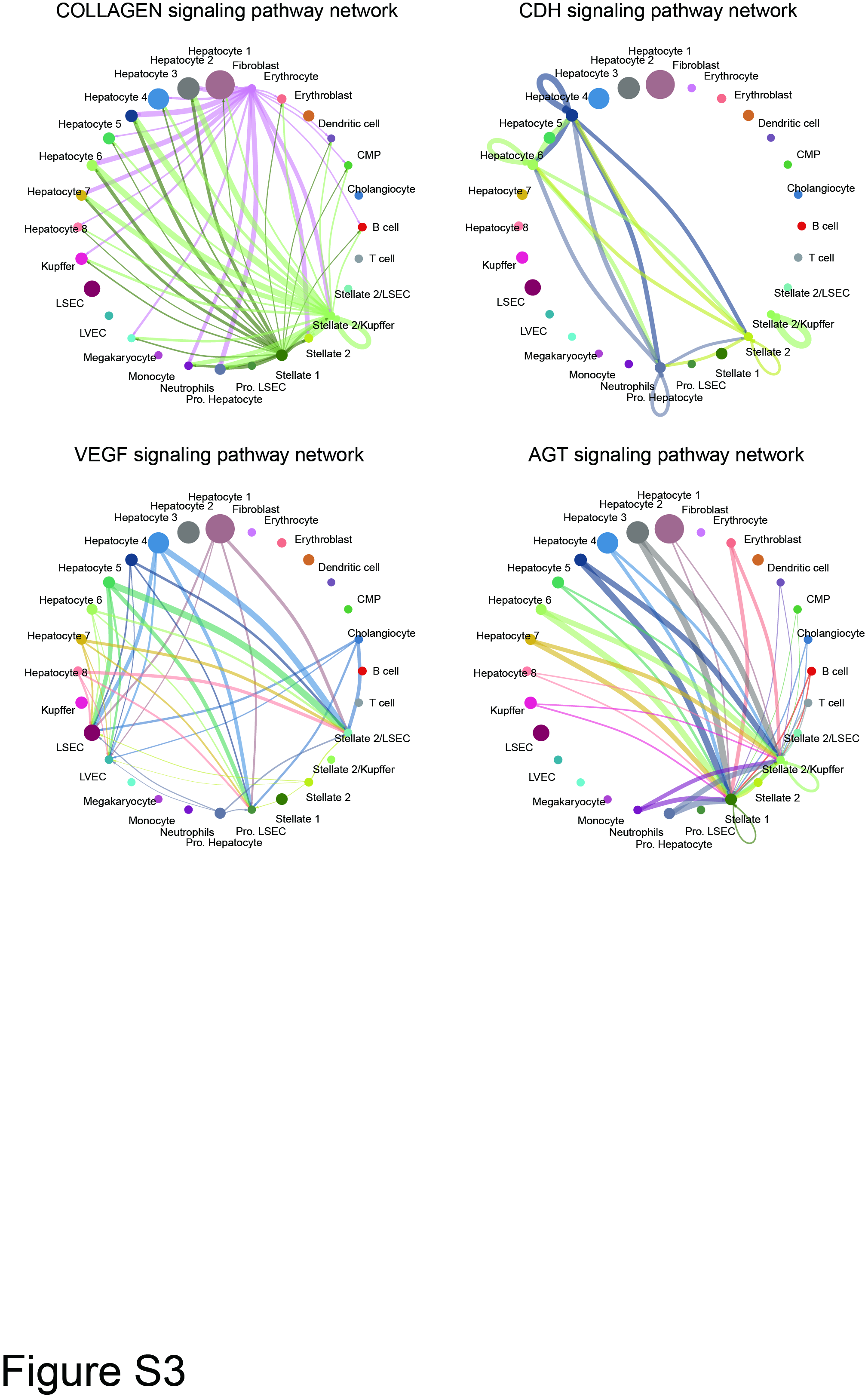

Supplement: Supplementary file 1 [file DataSheet1.ZIP › Additional files/FigureS3_noborder.tif]

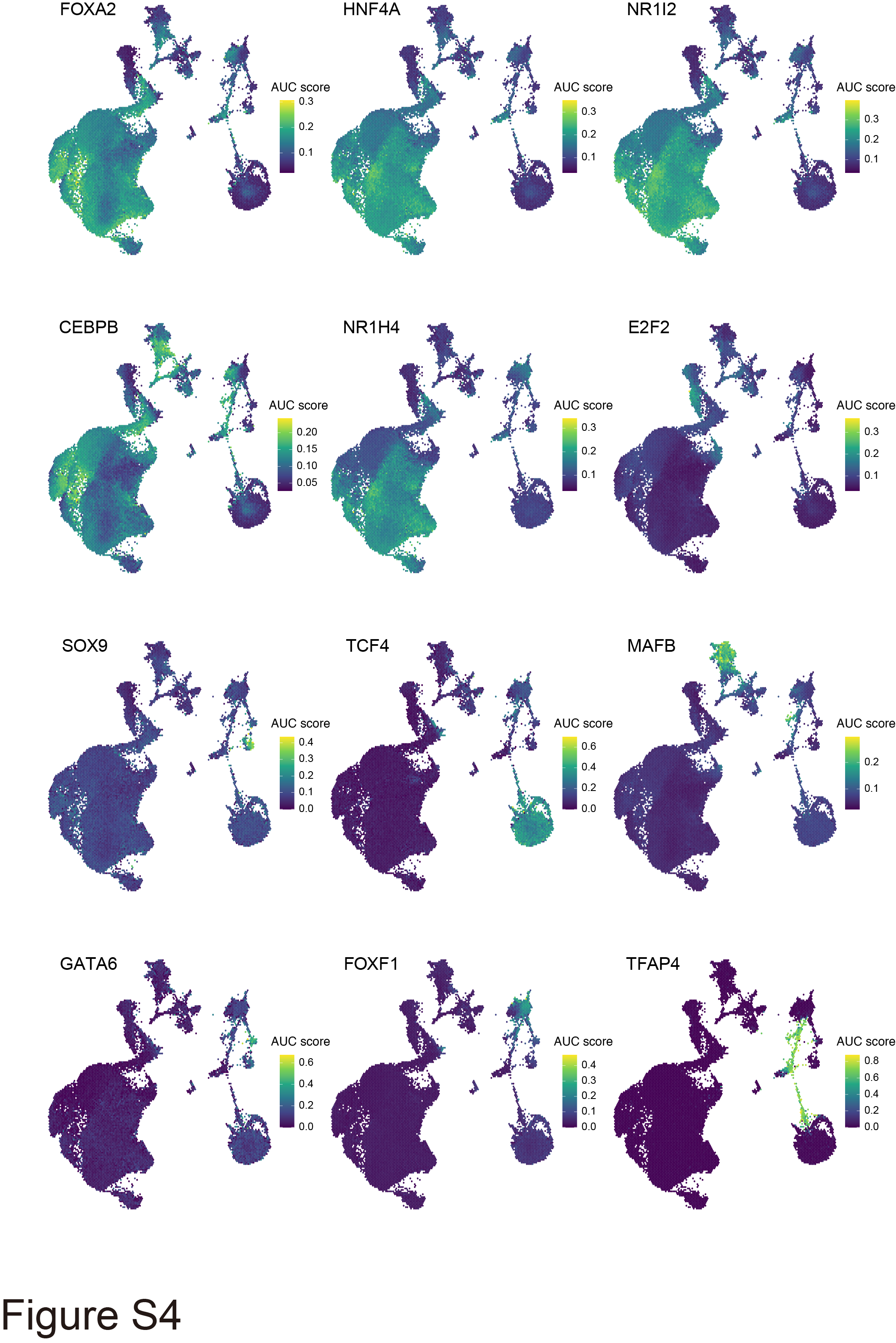

Supplement: Supplementary file 1 [file DataSheet1.ZIP › Additional files/FigureS4_noborder.tif]
